# Vagus Nerve Stimulation Increases Energy Expenditure: Relation to Brown Adipose Tissue Activity

**DOI:** 10.1371/journal.pone.0077221

**Published:** 2013-10-23

**Authors:** Guy H. E. J. Vijgen, Nicole D. Bouvy, Loes Leenen, Kim Rijkers, Erwin Cornips, Marian Majoie, Boudewijn Brans, Wouter D. van Marken Lichtenbelt

**Affiliations:** 1 Department of Human Biology, School for Nutrition, Toxicology and Metabolism – NUTRIM, Maastricht University Medical Center, Maastricht, The Netherlands; 2 Department of General Surgery, Maastricht University Medical Center, Maastricht, The Netherlands; 3 Department of Neurosurgery, Maastricht University Medical Center, Maastricht, The Netherlands; 4 Department of Nuclear Medicine, Maastricht University Medical Center, Maastricht, The Netherlands; 5 Epilepsy Center Kempenhaeghe, Heeze, The Netherlands; University of Warwick – Medical School, United Kingdom

## Abstract

**Background:**

Human brown adipose tissue (BAT) activity is inversely related to obesity and positively related to energy expenditure. BAT is highly innervated and it is suggested the vagus nerve mediates peripheral signals to the central nervous system, there connecting to sympathetic nerves that innervate BAT. Vagus nerve stimulation (VNS) is used for refractory epilepsy, but is also reported to generate weight loss. We hypothesize VNS increases energy expenditure by activating BAT.

**Methods and Findings:**

Fifteen patients with stable VNS therapy (age: 45±10yrs; body mass index; 25.2±3.5 kg/m^2^) were included between January 2011 and June 2012. Ten subjects were measured twice, once with active and once with inactivated VNS. Five other subjects were measured twice, once with active VNS at room temperature and once with active VNS under cold exposure in order to determine maximal cold-induced BAT activity. BAT activity was assessed by 18-Fluoro-Deoxy-Glucose-Positron-Emission-Tomography-and-Computed-Tomography. Basal metabolic rate (BMR) was significantly higher when VNS was turned on (mean change; +2.2%). Mean BAT activity was not significantly different between active VNS and inactive VNS (BAT SUV^Mean^; 0.55±0.25 versus 0.67±0.46, P = 0.619). However, the change in energy expenditure upon VNS intervention (On-Off) was significantly correlated to the change in BAT activity (r = 0.935, P<0.001).

**Conclusions:**

VNS significantly increases energy expenditure. The observed change in energy expenditure was significantly related to the change in BAT activity. This suggests a role for BAT in the VNS increase in energy expenditure. Chronic VNS may have a beneficial effect on the human energy balance that has potential application for weight management therapy.

**Trial Registration:**

The study was registered in the Clinical Trial Register under the ClinicalTrials.gov Identifier NCT01491282.

## Introduction

Obesity results from an imbalance between energy intake and energy expenditure and most applied obesity therapies are focussed on decreasing energy intake. [1]. However, in addition to a reduction in energy intake, increasing energy expenditure could be an effective means to prevent or treat obesity. In rodents, activated brown adipose tissue (BAT) is the main contributor to the regulated energy expenditure. [2]. Here, during cold exposure BAT activation increases energy expenditure by producing heat (thermogenesis) to prevent hypothermia. [2]. Recently functional brown adipose tissue (BAT) was also shown in adult man by means of FDG-PET-CT (^18^F-fluorodeoxyglucose positron-emission tomography and computed tomography). [3], [4]. Interestingly, BAT activity was inversely correlated with body mass index (BMI) and body fat percentage (BF%) [3], [5]. Moreover, subjects with active BAT show a significantly higher energy expenditure compared to subjects without BAT activity [5], [6]. This suggests BAT is related to the development of obesity and increasing BAT activity could be a new treatment modality for obesity. Rodent studies and histological analyses of human tissue samples have shown BAT is highly sympathetically innervated [7], [8]. Stimulation of sympathetic β-receptors on brown adipocytes was already suggested as a possible target for the treatment of obesity several years ago [9]. More recent studies report stimulation and suppression of BAT activity in rodents which is mediated through the vagus nerve [10], [11]. It is suggested the vagus nerve mediates peripheral signals to the central nervous system (CNS), which in turn connects to the sympathetic nerves that innervate BAT [11]. Interestingly, electrical stimulation of the cervical part of the vagus nerve (vagus nerve stimulation (VNS)) generates weight loss in rodents [12], [13]. Weight loss was also reported as a secondary outcome of VNS in humans, where it is used to treat refractory epilepsy [14], [15], [16], [17], [18], [19]. The weight loss induced by VNS may be the result of increased energy expenditure through BAT stimulation. However, most human trials are primarily focused on the effect of VNS on epilepsy from a neurological viewpoint and to date no prospective study has investigated the effect of VNS on energy expenditure. Therefore, this study aims to define the relation between VNS energy expenditure and BAT activation in a patient cohort on chronic stable VNS therapy for refractory epilepsy. We hypothesize that VNS increases energy expenditure by stimulating BAT activity.

## Methods

Approval for the study protocol was obtained from the ethics committee of the Maastricht University Medical Centre. The study was registered in the Clinical Trial Register under the ClinicalTrials.gov Identifier NCT01491282 (For the Study Protocol; see File S1). Between January 2011 and June 2012 written informed consent was obtained from 15 patients on stable VNS therapy using the Vagus Nerve Stimulation Therapy System (VNS Therapy®, Cyberonics Inc., Houston, Texas, USA) for refractory epilepsy. All included subjects had stable therapy (i.e. no recent adjustments in VNS settings or epilepsy medication (Table 1)) and were aged between 18 and 65 years. Exclusion criteria were daily epileptic seizures, pregnancy, ketogenic diet, mental retardation and psychological instability. Follow-up was completed in July 2012.

**Table 1:** Medication use per patient.

Subject	Medication
1	carbamazepine, lamotrigine, lacosamide
2	carbamazepine, clonazepam
3	levetiracetam, oxcarbazepine, clobazam
4	carbamazepine, valproic acid, lamotrigine, lacosamide
5	lamictal, levetiracetam, calciumcarbonate, domperidon, paracetamol, folic acid, hydroxychloroquin, bisacodyl, tramadol, zolpidem, pantoprazol, estradiol/norethisteron
6	gabapentine, clobazam, levetiracetam
7	levetiracetam, carbamazepine, desogestrel
8	clorazepine, gabapentine, topiramate
9	oxcarbazepine, calcium supplements
10	carbamazepine, lacosamide, L-carnitene
11	clobazam, retigabine, lacosamide, sertralin, desogestrel
12	levetiracetam, lamotrigine, clemastine, cinnarizine, omeprazol
13	pipamperon, lacosamide, clobazam, carbamazepine, levetiracetam
14	clobazam, lamotrigine, gabapentin, carbamazepine, perindopril/indapamide, dipyridamol/acetyl salicylic acid, bisoprolol, nifedipin, atorvastatin
15	fenytoin, clonazepam, oxcarbazepine

### Principle of vagus nerve stimulation

VNS is an approved procedure for refractory epilepsy patients. The therapeutic goal of VNS is a reduction in seizure frequency, seizure severity, and an improvement in general well-being. The VNS Therapy® System consists of a programmable pulse generator implanted subcutaneously at the level of the upper left chest. A helical bipolar electrode connected to the left vagus nerve transmits electric signals generated by the pulse generator. The stimulation parameters are adjusted via telemetric control to an optimal value for every individual patient. Standard starting parameters are: output current 0.25 mA, pulse width 250–500 µsec, frequency 30 Hz, On-period 30 sec, Off-period 5 min. If the effect is suboptimal, the output current is increased in steps of 0.25 mA to a maximum of 3 mA. The most common standard therapeutic setting is: output current 1–2 mA, pulse width 250 µsec, frequency 30 Hz, On-period 30 sec, Off-period 5 min. A second option is the introduction of Rapid Cycling, where the On-period is 7 sec and the subsequent Off-period 20 sec. Patients responding to Rapid Cycling are thought to need this extra stimulation to acutely suppress an upcoming seizure in order to obtain a reduction in their seizure frequency.

### Study protocol

Energy expenditure was measured using indirect calorimetry and BAT activity was assessed by means of FDG-PET-CT during actual VNS and when VNS was inactivated. This was possible since the anticonvulsive effect of VNS is considered chronic and short-term inactivation does not lead to an acute increase in seizures. In addition, we compared BAT activity during VNS and during mild cold stimulation. The mild cold intervention served as a control since it is known to activate BAT [3], [5]. Ideally, one would like to compare the effect of active/inactive VNS with and without cold exposure, which would result in four FDG-PET-CT measurements in each subject. However, due to ethical considerations a maximum of two FDG-PET-CT measurements was allowed. Therefore, ten patients were measured in thermoneutral (TN) conditions with active (VNS-On) and inactive VNS (VNS-Off) respectively. In addition, five subjects were measured with active VNS in TN conditions (VNS-TN) and during mild cold exposure (VNS-Cold) respectively (Table 2).

**Table 2:** Subject characteristics for all subjects, the intervention group with Vagus Nerve Stimulator (VNS) On and Off (n = 10) and for the group with VNS during thermoneutral (VNS-TN) conditions and cold exposure (VNS-Cold) (n = 5).

Characteristics	Group	VNS-On/Off	VNS- TN/Cold	P-value
n	15	10	5	
Sex (Male/Female)	10 M/5 F	4 M/6 F	1 M/4 F	
Age (yrs)	45±10	42±10	49±8	0.203
Height (cm)	170±10.0	168.5±8.4	173±13	0.464
Mass (kg)	73.0±11.6	70.1±11.7	78.6±10.4	0.194
BMI (kg/m^2^)	25.2±3.5	24.6±3.0	26.6±4.4	0.316
Fat Free Mass (kg)	49.8±9.0	48.8±9.5	51.7±8.5	0.568
Fat Mass (kg)	21.9±6.4	20.4±5.3	25.0±8.0	0.199
Body Fat (%)	29.5±6.9	28.5±6.4	31.4±8.2	0.459
VNS output current (mA)	1.58±0.55	1.55±0.59	1.65±0.52	0.752
VNS implant time (months)	59±19	64±15	50±24	0.185
VNS implant mass (kg)	71.2±12.5	69.8±13.9	72.8±12.0	0.717
VNS implant BMI (kg/m^2^)	24.7±3.4	24.9±3.1	24.5±4.1	0.861

P-values shown for unpaired t-tests between VNS-On/Off and VNS-TN/Cold. * P<0.05.

Subjects were measured under fasted conditions (no food intake from 10 p.m. the night before, only water consumption was allowed) from 9 a.m. to 2 p.m. under supervision of a specialized research nurse (L.L.). VNS-On and VNS-Off took place on separate occasions within 14 days. During VNS-Off the system was inactivated (output current 0 mA, magnet function 0 mA) at 9:30 a.m. prior to the measurements. At the end of the test day (2:00 p.m.) the VNS system was re-activated. During VNS-Cold the settings of the VNS system were not adjusted. VNS-TN and VNS-Cold were also performed within a 14-day period. Body composition (body fat%, fat mass (FM), fat free mass (FFM)) was determined by dual x-ray absorptiometry (DXA, type Discovery A, Hologic, Bedford, MA, USA).

During the experiments the subjects wore standardized clothing (socks 0.02 clo (clothing insulation factor), shirt 0.09 clo, sweatpants 0.28 clo, underwear 0.04 clo, total clo factor 0.43 clo) [20]. A catheter was placed in the left antecubital vein for blood sampling during measurements and to inject ^18^F-Fluoro-Deoxy-Glucose (FDG) for FDG-PET-CT (for further detail on scanning protocol see below). On every test day baseline plasma glucose values were determined to rule out hyper- or hypoglycaemia. All subjects had normal plasma glucose values that did not differ between measurements (VNS-On/Off; 5.1±0.5 versus 5.2±0.5 mmol/L, P = 0.369, VNS-TN/Cold; 5.2±0.7 versus 4.9±0.5 mmol/L, P = 0.881). Body core temperature was monitored by a telemetric pill that was orally ingested (CoreTemp, HQ Inc., Palmetto, FL, USA). iButtons were placed at 14 ISO-defined skin sites to measure skin temperatures [21]. For comparison between proximal and distal body temperatures, the proximal skin temperature was defined as the mean of the iButtons placed on the chest, abdomen, shoulder and lower back. The distal skin temperature was defined as the mean of the iButtons placed on the wrist and foot. Next, subjects were placed in a semi-supine position on a nephrodialysis chair covered with a water-perfused mattress (necessary for the cold exposure protocol, see below) in a specially equipped tent (Colorado Altitude Training, Louisville, CO, USA) where energy expenditure was measured by indirect calorimetry using a ventilated hood system (Omnical, Maastricht Instruments BV, Maastricht, the Netherlands). Laser Doppler Flowmetry (PF5000; Perimed, Jarfalla, Sweden) was used to determine skin blood flow gradients dorsal of the medial malleolus and at the glabrous skin of the ventral hand thenar to monitor whether a subject was in its thermoneutral zone as reported [22]. The VNS-On/Off (n = 10) and VNS-TN (n = 5) measurements were performed under thermoneutral conditions (average room temperature VNS-On; 24.9±0.8°C, VNS-Off; 25.2±1.0°C, VNS-TN; 25.2±0.9°C, P = 0.7260, for details on thermoneutral conditions see Kingma et al. [22]). After the first hour of measuring under thermoneutral conditions (t0–t60) 10 ml FDG was injected. After the second hour (t60–t120), FDG-PET-CT imaging was performed to quantify metabolically active BAT. The scanning protocol, FDG activity (74 MBq) and data analysis were identical to our previous studies on BAT activity [5], [23]. During VNS-Cold a personal cooling protocol was used as described earlier in detail [5]. In short, after measuring under thermoneutral conditions (t0–t30), the subject was cooled until they reported shivering. Upon the first signs of shivering the temperature was raised slightly (on average 2.3±0.6°C) to ensure only non-shivering thermogenesis (NST) [5]. After one hour of stable cooling situation (t30–t90), 10ml FDG was injected, followed by the second hour of cooling (t90–t150) and subsequent FDG-PET-CT imaging.

### FDG-PET-CT analysis

**Figure 1:**
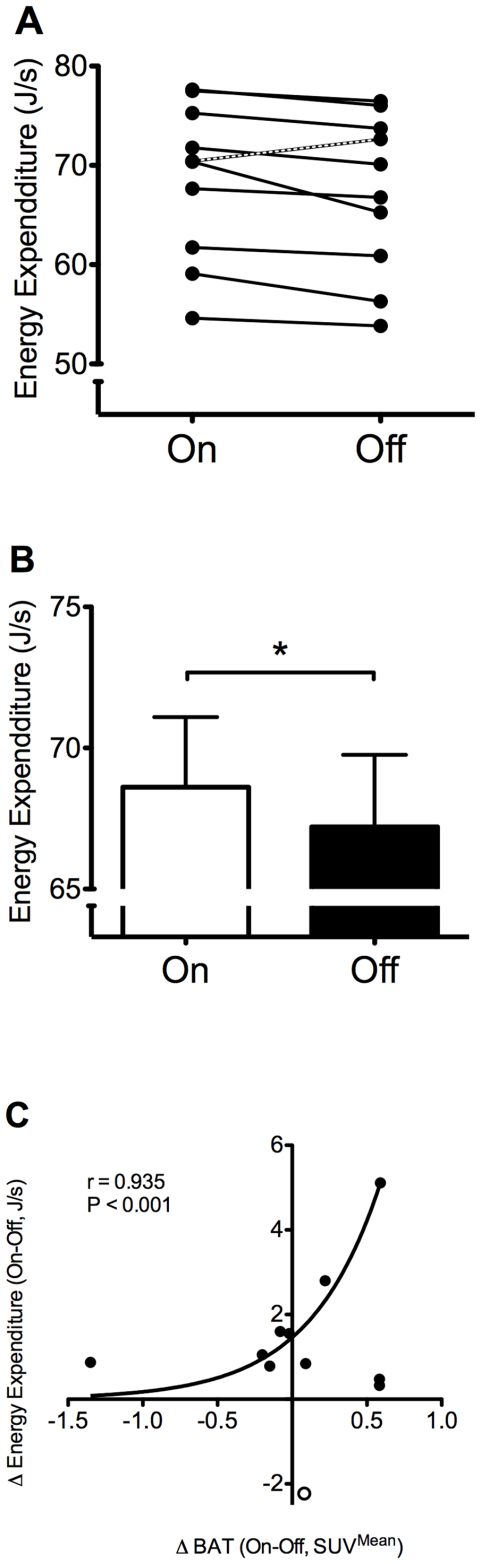
Basal metabolic rate (BMR) during active and inactive VNS in relation to BAT activity. **A**. Individual responses upon VNS intervention. Notice all but one (marked line) subjects decreased energy expenditure upon deactivation of the VNS (subject that increased energy expenditure also described in Results section). **B**. Mean group BMR upon VNS intervention. **C.** The change in energy expenditure (Δ Energy Expenditure) upon VNS intervention (On/Off) correlated to the change in BAT activity. One subject (open dot) showed a high increase in energy expenditure upon VNS intervention. This subject was confirmed as an statistical outlier in the exponential growth equation shown and therefore excluded from the equation. Values shown are means + S.E.M. Significance shown for paired t-test in VNS intervention group consisting of 4 males and six females. * P<0.05

The analysis of PET-CT images was similar to Vosselman et al [24]. For comparison, standard cube volumes-of-interest (VOIs) were placed in the tissue type studied: Brown Adipose Tissue in the supraclavicular region (BAT), deltoid muscle (Deltoid), biceps muscle (Biceps), triceps muscle (Triceps), erector spinae muscle at the level of vertebrae C7 (C7), T8 (T8), and L3 (L3), the mean of all analyzed muscles (Muscle), subcutaneous white adipose tissue in the dorsolumbar region near vertebrae L-3 and L-4 (WAT Sc), visceral white adipose tissue (WAT Visc), the liver (Liver) and the brain at the level of the cerebellum (Brain) (Figure 1). The volume of these three-dimensional cubes was 1.33 cm^3^ (i.e. three consecutive slices of 0.44 cm^3^ thickness). For BAT the placement of one cube in supraclavicular adipose tissue often interferes with adjacent muscle and hence the cube volume in BAT was composed of three separate slices of 1.0×1.0×0.44 cm. When drawing the cubes, attention was paid to the Hounsfield Units (HU) on CT to define the specific tissue type and to avoid interference with adjacent tissue. For example, adipose tissue has a HU of −200 to −10 and muscle 10 to 100. Tissue activity on FDG-PET-CT was expressed in standard uptake value (SUV; as calculated by uptake (kilobequerels per ml) per injected dose (kilobequerels) per patient weight (grams)) using the PMOD Biomedical Image Quantification tool (PMOD Technologies Ltd., Zurich, Switzerland). Tissue activity of each region was determined by the mean SUV uptake (SUV^Mean^). Finally, FDG-PET-CT imaging during the Off-measurement failed in one subject due to a critical image reconstruction error. Consequently, values shown are for nine subjects in the VNS-On/Off group and five subjects in the VNS-TN/Cold group.

### Statistics

Reported data were expressed as means ± S.D. Statistical analyses were performed with PASW Statistics 18.0 for Mac OS X 10.6.4. Repeated measurements (VNS-On versus VNS-Off, and VNS-TN versus VNS-Cold) were compared using paired student's t tests. Comparisons between groups (VNS-On/Off versus VNS-TN/Cold) were made using one-way ANOVA with a post-hoc Bonferroni correction. A P-value below 0.05 was considered significant.

## Results

### Subject characteristics

Ten male and five female patients with a mean age of 45±10 years and a mean Body Mass Index (BMI) of 25.2±3.5 kg/m^2^ that were (successfully) treated with VNS for refractory epilepsy were included (Table 2). VNS implantation was on average 59±19 months (range; 22–89 months) ago and all subjects did not have any recent adjustments in their VNS settings or medication (last adjustment >1 month ago, VNS output current; 1.58±0.55 mA, Table 2, for medication see Table 1). In this way we aimed to rule out the influence of medication or VNS adjustments in the best possible way in this specific patient cohort. Pre-VNS treatment body weight and BMI were retraceable for 11 subjects and were not significantly different from weight and BMI during the study (implant weight and BMI; 71.2±12.5 kg, 24.7±3.4 kg/m^2^, current weight and BMI; 72.9±11.6 kg, 25.2±3.5 kg/m^2^, P = 0.414). The subject characteristics were not different for the On/Off (n = 10) versus the TN/Cold group (n = 5) (Table 2).

### Energy expenditure

Basal metabolic rate (BMR) decreased significantly when VNS was turned off (68.6±7.9 J/s versus 67.2±8.1 J/s, P = 0.038, mean change; 2.2%, range; −3.1 to 7.8%, Table 3, Figure 1). All but one subject decreased their BMR; the male subject with increased BMR was very restless during the Off-measurement due to personal affairs. Exclusion of this subject revealed energy expenditures of 68.6±7.9 J/s (VNS-On) and 66.6±8.31 J/s (VNS-Off) (P = 0.005). The subject's data remained included in the further presented analyses.

**Table 3:** Energy expenditure, core and skin temperatures, skin perfusion and Standard Uptake Values (SUV^Mean^) for Brown Adipose Tissue (BAT), all muscles analyzed (Muscle), deltoid muscle (Deltoid), biceps muscle (Biceps), triceps muscle (Triceps), erector spinae muscle at the level of vertebrae C7 (C7), T8 (T8), L3 (L3), subcutaneous white adipose tissue (WAT Sc), visceral white adipose tissue (WAT Visc), the liver (Liver) and the brain at the level of the cerebellum (Brain).

Parameter	On	Off	P-value	TN	Cold	P-value
Energy expenditure (J/s)	68.6±7.9	67.2±8.1	0.038*	68.2±1.5	75.1±7.7	0.107
Core temperature (°C)	37.0±0.3	36.9±0.4	0.738	36.9±0.2	36.9±0.23	0.877
Mean skin temperature (°C)	33.5±0.8	33.6±0.7	0.686	33.2±0.4	29.0±0.8	<0.001**
Proximal skin temperature (°C)	34.6±1.1	34.6±0.5	0.996	34.0±1.3	31.1±0.8	0.012*
Distal skin temperature (°C)	31.7±1.6	33.0±1.7	0.016*	31.7±2.5	24.0±1.5	<0.001**
Perfusion Foot (AU)	8.2±3.9	13.4±21.4	0.396	9.6±8.0	6.8±4.4	0.553
Perfusion Hand (AU)	54.5±35.3	81.7±70.5	0.149	25.1±11.4	6.0±1.4	0.029*
SUV^Mean^ BAT	0.55±0.25	0.67±0.46	0.619	0.65±0.29	3.40±1.63	0.012*
SUV^Mean^ Muscle	0.57±0.14	0.65±0.11	0.100	0.78±0.14	0.72±0.14	0.012*
SUV^Mean^ Deltoid	0.57±0.16	0.62±0.10	0.281	0.74±0.11	0.65±0.14	0.031*
SUV^Mean^ Biceps	0.62±0.17	0.69±0.17	0.390	0.89±0.14	0.81±0.19	0.130
SUV^Mean^ Triceps	0.51±0.14	0.61±0.13	0.024*	0.70±0.16	0.77±0.07	0.260
SUV^Mean^ C7	0.62±0.22	0.66±0.13	0.572	0.85±0.23	1.19±0.53	0.209
SUV^Mean^ T8	0.63±0.12	0.72±0.10	0.081	0.79±0.16	0.84±0.13	0.475
SUV^Mean^ L3	0.46±0.15	0.60±0.16	0.057	0.75±0.29	0.63±0.07	0.389
SUV^Mean^ WAT Sc	0.19±0.09	0.24±0.08	0.194	0.24±0.09	0.23±0.09	0.742
SUV^Mean^ WAT Visc	0.36±0.16	0.55±0.30	0.111	0.47±0.13	0.49±0.32	0.881
SUV^Mean^ Liver	1.60±0.46	2.04±0.45	0.038*	2.44±0.41	2.33±0.41	0.405
SUV^Mean^ Brain	6.58±2.07	6.82±1.65	0.730	7.45±1.68	8.02±1.45	0.183

AU denotes Arbitrary Units for skin perfusion. * P<0.05, ** P<0.001.

Upon cold exposure BMR increased as compared to thermoneutral conditions (TN; 68.2±1.5 J/s versus Cold; 75.1±7.7 J/s, P = 0.107, Table 3). BMR in the VNS-TN group was similar to BMR in the VNS-On group (P = 0.919).

### Core and skin temperatures

Body core and mean skin temperatures did not change significantly when VNS was turned off (core temperature; 37.0±0.3°C versus 36.9±0.4°C, mean skin temperature; 33.5±0.8°C versus 33.6±0.7°C, P = 0.738, Table 3). When skin temperatures were divided in proximal and distal zones [21], the distal skin temperature was significantly increased during the Off-measurement (31.7±1.6°C versus 33.0±1.7°C, P = 0.016, Table 3).

During cold exposure skin temperatures decreased significantly (TN: mean skin temperature; 33.2±0.4°C versus Cold: 29.0±0.8°C, P<0.001, Table 3).

### Skin perfusion

Perfusion of the medial foot and ventral hand did not change during VNS (foot; 8.2±3.9 Arbitrary Units (AU) versus 13.4±21.4 AU, P = 0.396, hand; 54.5±35.3 AU versus 81.7±70.5 AU, P = 0.149, Table 3). Cold exposure significantly decreased hand but not foot perfusion (hand; 25.1±11.4 AU versus 6.0±1.4 AU, P = 0.029, foot; 9.6±8.0 AU versus 6.8±4.4 AU, P = 0.396, Table 3).

### BAT activity and FDG uptake in other tissues

**Figure 2:**
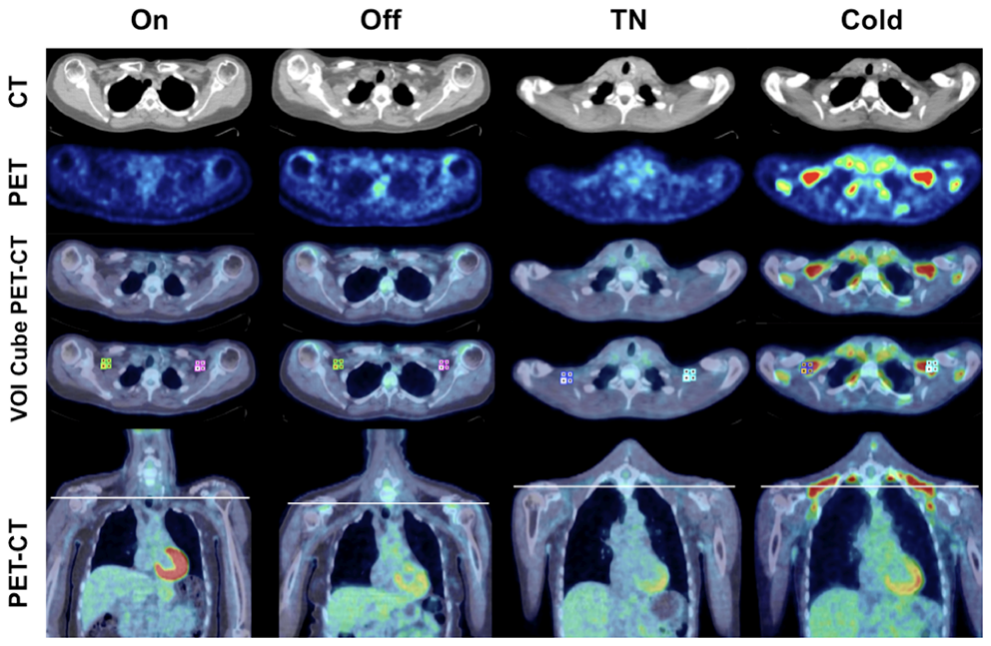
FDG-PET-CT images of intervention group and cold exposed subjects. Transaxial and coronal CT, PET and PET-CT fused images of VNS-On and VNS-Off from a subject in the VNS-On/Off group and images from a subject in the VNS-TN/Cold group during thermoneutral conditions (TN) and cold exposure (Cold). PET-CT image shown with and without Volume-Of-Interest (VOI) cubes for determination of activity. The white line in the coronal image indicates the transaxial slice shown above.

**Figure 3:**
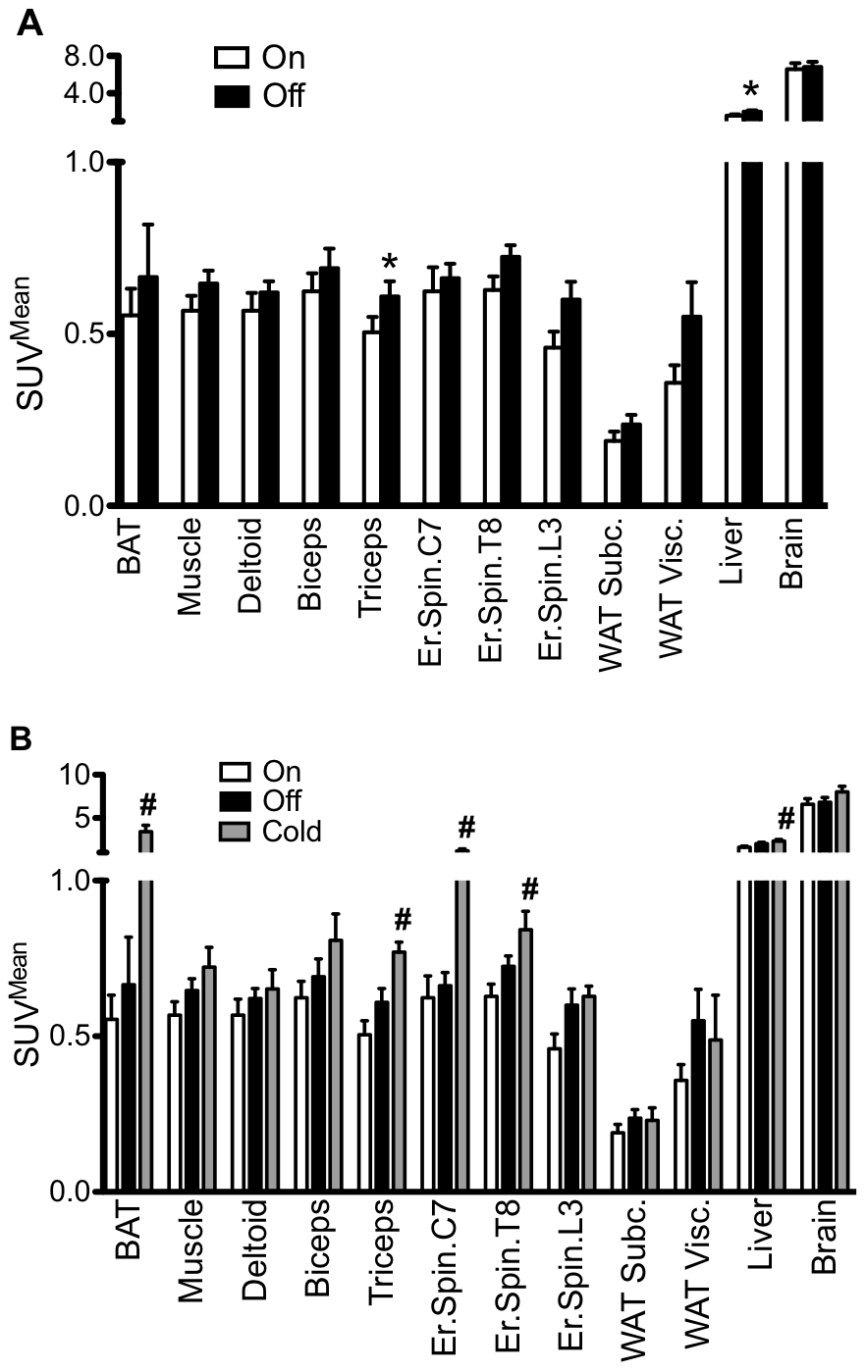
FDG-PET-CT activity of different tissue types upon VNS intervention. SUV^Mean^ values for Brown Adipose Tissue (BAT), all muscle (Muscle), deltoid muscle (Deltoid), biceps muscle (Biceps), triceps muscle (Triceps), erector spinae muscle at the level of vertebrae C7 (C7), T8 (T8), L3 (L3), subcutaneous white adipose tissue (WAT Sc), visceral white adipose tissue (WAT Visc), the liver (Liver) and the brain at the level of the cerebellum (Brain). **A.** Comparison of activity during VNS-On and VNS-Off. **B.** Comparison of activity between VNS-On, VNS-Off and VNS-Cold. Values shown are mean + S.E.M. * P<0.05 for paired t-tests between VNS-On and VNS-Off. # P<0.05 for one-way ANOVA with post-hoc Bonferroni correction between VNS-On, VNS-Off and VNS-Cold.

Figure 2 shows representative images of FDG-uptake on PET-CT in the studied groups. The mean SUV for BAT showed no statistical difference during VNS (BAT SUV^Mean^; 0.55±0.25 versus 0.67±0.46, P = 0.619, Table 3, Figure 3). In different muscles analyzed, the triceps muscle had a significantly increased FDG-uptake when VNS was turned off (Triceps SUV^Mean^; 0.51±0.14 versus 0.70±0.16, P = 0.024, Table 3). However, for all muscles together there was no significant change in activity (Muscle SUV^Mean^; 0.57±0.14 versus 0.65±0.11, P = 0.100, Table 3). The FDG-uptake in the liver significantly increased during VNS (Liver SUV^Mean^; 1.60±0.46 versus 2.04±0.45, P = 0.038, Table 3). After cold exposure, all subjects showed increased BAT activity (BAT SUV^Mean^; 0.65±0.29 versus 3.40±1.63, P = 0.012, Table 3, Figure 3). Deltoid muscle activity was significantly decreased in the cold (Deltoid SUV^Mean^; 0,74±0.11 versus 0.65±0.14, P = 0.031, Table 3, Figure 3) and also activity for all muscles together decreased upon cold exposure (Muscle SUV^Mean^; 0.78±0.14 versus 0.72±0.14, P = 0.012, Table 3, Figure 3).

### Correlations

Energy expenditure during VNS-On and VNS-Off measurements was not related to BAT activity, activity of other tissue (Muscle, WAT), skin perfusion, core and skin temperatures or any other study parameter in either uni- or multivariate analyses. However, the change in energy expenditure upon VNS intervention (from VNS-On to VNS-Off) was positively correlated to the change in BAT activity (exponential curve fitting, r = 0.935, P<0.001). The change in energy expenditure from the subject that was restless during the VNS off measurement was considered as an outlier, because of Dixon's Q-test for small sets of replicate observations revealed that this was a significant outlier (Q = 1.0%). The result from this subject is indicated in Figure 1 by an open symbol. VNS output current was not related to BAT activity or energy expenditure.

## Discussion

This study shows that even short-term interruption of VNS therapy by turning off the VNS for only several hours significantly decreased energy expenditure in a cohort of treatment-stable VNS patients. In addition, the change in energy expenditure upon inactivation of VNS was significantly related to a change in BAT activity. Therefore, VNS possibly positively influences energy balance by increasing energy expenditure through involvement of BAT in humans. On top of that, the parasympathetic VNS induced thermogenesis suggests that VNS affects sympathetic mechanisms. This indicates a functional link between the parasympathetic and sympathetic nervous system, as further discussed below.

### Mechanism of VNS increased energy expenditure

Possible mechanisms by which VNS affects energy expenditure have been suggested in several studies. From an anatomical viewpoint, it is interesting to note that the majority of fibers present within the vagus nerve are afferent fibres (74%) and only a minority (26%) are efferent, indicating that the vagus nerve is both an afferent nerve and efferent nerve as well [25].

Within the hypothalamus, several areas have been studied in respect to the regulation of energy balance. The hypothalamic arcuate nucleus receives vagus signals and projects them to the paraventricular nucleus (PVN), which in turn signals to the sympathetic nervous system and to the thyroid axis [26]. Vagotomy blocks these responses and alters energy homeostasis [26]. In the ventromedial nucleus of the hypothalamus (VMH), an area adjacent to the PVN, both parasympathetic and sympathetic neurons are present, and it is suggested the VMH regulates the ‘balance’ between sympathetic and parasympathetic nerve activities. Therefore, VMH stimulation may affect sympathetic mechanisms. Electrical stimulation of the VMH increased energy expenditure and decreased RQ in rats, suggesting an increased fat oxidation [27]. Indeed, VMH stimulation, increases norepinephrine turnover and thermogenesis in interscapular BAT in rats, and this effect is completely inhibited by blockage of sympathetic ganglions [28], [29]. Several studies have addressed the effect of vagotomy on BAT activity. Saindon et al. found no effect of a cervical vagatomy on BAT activity,[30] whereas another report shows a decrease in both WAT and BAT weight after a subdiaphragmatic vagatomy in obese rats [31]. Interestingly, vagotomy has been demonstrated to decrease NE-turnover in BAT,[11] suggesting that vagus signals may influence BAT thermogenesis. Specific stimulation of the central nervous system though injection of enterostatin, a pancreas released peptide known to reduce food intake, increased sympathetic BAT activity [32], [33]. Enterostatin signalling is known to be dependent on an intact vagus nerve, since vagatomy inhibits its effect [32]. In summary, sympathetically induced BAT activity seems to be at least partially influenced by parasympathetic vagal signalling.

If VNS induces a VMH-mediated increase in sympathetically induced BAT activity, the mechanism for VNS-induced BAT stimulation would then be different from physiological BAT stimulation by cold sensation, which is by peripheral nerves and proceeds superiorly to the brain via the spinal cord rather than the vagus nerve. Therefore, physiological cold-induced BAT activity could be mediated differently and possibly vagus mediated signals could also be part of other mechanism that stimulate BAT such as diet-induced thermogenesis (DIT) which was shown recently by Vosselman et al. [24]. However, the current human report is limited from measuring activity in specific parts of the spinal cord and the brain. Therefore, to exactly investigate the pathway of DIT and vagal afferents possibly animal studies should be performed.

In summary, in the present study we observed a significant change in energy expenditure that was related to changes in the activity in BAT and not to changes in any of the other tissues that we have measured. Despite the fact that mean BAT activity did not increase upon VNS, the change in BAT activity explained a significant part of the change in energy expenditure. To our opinion, this suggests at least part of the effect of VNS intervention on energy expenditure can be explained by BAT activity.

### Comparison with previous studies on VNS therapy

An effect of VNS on metabolism had already been suggested by the authors of a brief report where weight loss was observed in 17/27 patients with VNS for epilepsy (2002) [17]. Pardo et al. studied a morbidly obese cohort of 14 VNS patients treated for depression (weight 91±27 kg, BMI 43±5 kg/m^2^) with VNS settings comparable to the present study (>24 months VNS implant, Output current; 0.25–1.5 mA, Freq; 30 Hz, Pulse; 500-μs, On-period; 30 s, Off-period; 5 min, VNS current study; Output current; 1.58±0.55 mA, Freq; 30±0 Hz, Pulse; 266.7±64.5 μs, On-period; 23.9±10.5s, Off-period; 3.7±2.1min) and observed a significant BMI-related weight loss after VNS therapy[16]. The short-term intervention in normal weight subjects in the current study already showed a significantly increased energy expenditure during VNS. Although the mean difference in BMR was small, the weight loss observed by Pardo et al. in morbidly obese subjects suggests long-term VNS results in increased energy expenditure and could be important in weight maintenance.

Another study reported 33 obese patients (BMI; 30.1±3.6 kg/m^2^) receiving VNS for depression and investigated the response to a food stimulus upon active and inactive VNS [19]. Short-term VNS intervention significantly altered food craving. Duration of VNS intervention was not specified, but seems to have taken place within several hours, which is similar to the present study [19].

All currently published data on the effect of VNS on metabolism in humans are retrospective. However, in animals a prospective study in overfed minipigs undergoing bilateral VNS (implanted 5 cm above the diaphragm) with stimulation parameters comparable to our study (VNS minipigs; Output current; 2 mA, Freq; 30 Hz, Pulse; 500-μs, On-period; 30 s, Off-period; 5 min, VNS current study; see above) has revealed that weight gain was prevented in the VNS-treated minipigs and not in controls [34]. VNS in these minipigs significantly decreased food intake, especially carbohydrate consumption (‘sweet craving’) as assessed by the respiratory quotient (RQ) [34]. In the present study we did not observe a difference in RQ after VNS intervention (RQ On; 0.82±0.03 versus RQ Off; 0.82±0.04, P = 0.8774) and the mean RQ of±0.8 confirms the prescribed overnight fast preceeding the BMR measurments. However, possibly RQ does show a significant difference in man when studying VNS in longer time periods, for example before and after VNS implantation.

Finally, in a study on the effect of VNS on metabolism Sobocki et al. used a crossover design in pigs with activation and de-activation, each in a 4-week period and found that VNS prevented weight gain but did not affect metabolic rate as assessed by indirect calorimetry [35]. However, metabolic rate was determined under general anesthesia, which probably affected BMR measurements. Possibly BMR did change under awake conditions, as was observed in the current report, which would explain the significant differences in weight gain observed by Sobocki et al.

### BAT presence and activity in refractory epilepsy

Since BAT activity is reported to decrease with age, [36] can be affected by several pathophysiological conditions, [37] and could be influenced by medication such as beta-blockers [38], [39]. we considered the possibility of reduced presence of BAT in (these) patients with refractory epilepsy. Therefore, we investigated maximal BAT activity in VNS patients after mild cold exposure. Mild cold exposure is known to induce BAT activation in 100% of lean young subjects (age; 24.3±3.6 years, BMI; 23.2±1.2 kg/m^2^) and to be related to BMR and cold-induced thermogenesis [3], [5], [6]. By using our personalized cooling protocol we prevented shivering and aimed for the effect of maximal non-shivering thermogenesis on BAT activity. This excludes a possible effect of shivering on vagally mediated BAT activity. Despite their relatively high mean age (49±8 years) but comparable BMI (26.6±4.4 kg/m^2^), cold-induced BAT activity was present in all five patients. Cold stimulated BAT presence and activity were significantly higher than under TN conditions (VNS-Cold; BAT SUV^Mean^ 3.40±1.63 versus VNS-TN; BAT SUV^Mean^ 0.65±0.29, P = 0.012), but slightly lower as compared to previously studied healthy young subjects (Age; 23±2 years, BMI; 23.1±1.7 kg/m^2^, BAT SUV^Mean^; 7.19±2.09 versus current study BAT SUV^Mean^; 3.40±1.63, P = 0.010, Vosselman et al., unpublished data). We believe this may be attributed to an age-related decrease in BAT activity [36]. In conclusion, BAT presence and activity thus does not appear to be decreased in patients with refractory epilepsy.

### Effect of VNS on muscle and liver metabolism

In contrast to the significant increase in energy expenditure that was related to BAT activity, we observed a decrease in liver and triceps muscle glucose uptake on FDG-PET-CT upon VNS. Possibly, the activity in liver and muscle could be dependant on other substrates than glucose. For example, it is known increased fatty acid oxidation can lead to inhibition of glucose uptake (the so-called Randle effect), [40]. which would decrease activity on FDG-PET-CT imaging since this selectively depicts glucose uptake. Therefore, although there is a decrease in FDG-PET-CT-activity in liver and muscle, there could still be increased metabolism upon VNS in these tissues resulting from increased fatty acid oxidation. However, in this study we were not able to study fatty acid metabolism since FDG-PET-CT is a glucose tracer. In this regard, more research focused on liver and muscle function as well as fatty acid metabolism upon VNS is needed.

### Limitations

This study has several limitations. First, the included patient group was relatively small. The number of patients with VNS therapy in our clinic is limited and we aimed for inclusion of a stable treatment cohort with no recent adjustments in their medication to exclude a medication effect on energy expenditure and BAT activity. Based on previous studies, we calculated inclusion of the current number of patients would be sufficient to detect possible differences in energy expenditure and BAT activity. Second, we were not able to obtain additional evidence of the balance between sympathetic and parasympathetic in the form of e.g. heart rate, blood pressure and plasma catecholamines, since blood sampling was limited and we did want to disturb during energy expenditure measurements.

Finally, although the difference in energy expenditure was significant, no difference was observed in BAT activity during VNS On and Off measurements. However, the changes in energy expenditure and BAT activity were related. Probably, the relatively short intervention period did not induce potential larger differences in BAT activity. Therefore, future studies should aim for longer intervention periods, preferably in a prospective manner.

## Conclusion

The present study demonstrates for the first time that VNS is accompanied by an increase in whole body energy expenditure, and secondly that this VNS thermogenesis is related to changes in BAT activity. The results therefore suggest that parasympathetic VNS induces a sympathetic thermogenic response that in its turn activates BAT. Possibly, specific hypothalamic areas are responsible for the regulation of this nervous system balance. Chronic VNS might have a beneficial effect on energy balance and might play a role in weight management. However, the observed mean differences in energy expenditure are small, probably to a large extent because of the short duration of the intervention. Future, long-term, prospective studies should point out if VNS has a clinically and therapeutically significant effect on BAT activity and whole body metabolism. In addition, molecular studies should assess the effect of VNS on the regulation of the sympathetic nervous system and the areas in the brain that could be involved.
